# From Policy to Plate: Implications of 2025 U.S. Federal Policy Changes on School Meals

**DOI:** 10.3390/nu17233696

**Published:** 2025-11-25

**Authors:** Lindsey Reed, Megan Elsener Lott, Mary Story

**Affiliations:** Healthy Eating Research, Duke University, Durham, NC 27710, USA; megan.lott@duke.edu (M.E.L.); mary.story@duke.edu (M.S.)

**Keywords:** National School Lunch Program, School Breakfast Program, free and reduced-price lunch, direct certification, school-age children, U.S. food policy

## Abstract

School meals are a cornerstone of the United States’ nutrition safety net for children from low-income families, providing nearly 30 million lunches daily. However, recent U.S. policy actions may limit access to school meals for children who need them most. The One Big Beautiful Bill Act changed eligibility requirements to limit participation in the Supplemental Nutrition Assistance Program (SNAP) and Medicaid. School-age children enrolled in SNAP and Medicaid are automatically enrolled in school meal programs. Limiting participation in SNAP and Medicaid has the potential to significantly impact children’s ability to receive nutritious school meals at low or no cost, will make it harder for schools to participate in community eligibility provision, and will increase the administrative burden on school staff and parents.

## 1. Introduction

### 1.1. Current Reach and Participation

The School Breakfast Program (SBP) and National School Lunch Program (NSLP) are cornerstones of the U.S. nutrition safety net, serving millions of children each day. During the 2023–2024 school year, the SBP served breakfast to 15.4 million children and the NSLP served lunches to 29.4 million children daily across the United States [1]. Of the nearly 30 million lunches served daily, 20.1 million children received free meals, 946,257 received reduced-price meals, and 8.3 million received a paid lunch [2].

Participation also differs by grade level, race and ethnicity, household income, and urban-rural school setting. A majority (71%) of U.S. elementary school students participate in the Federal school lunch program, compared to 52% of middle school students and 39% of high school students [3]. Across both breakfast and lunch programs, participation is highest among Hispanic and Black students and students from lower-income households [3]. In school year (SY) 2022–2023, 87% of public schools participated in the SBP and 91% participated in the NSLP [2], with roughly one-third (34.9%) of schools participating in the NSLP located in rural areas. On average, school meal participation rates are higher in rural schools compared to urban schools [3,4].

### 1.2. Policy Context and Improvements

School meals play a critical role in child health and development. Children in the U.S. may receive up to half of their daily caloric intake at school, and school meals remain one of the healthiest food sources for school-aged children [5]. The federal Healthy, Hunger-Free Kids Act (HHFKA) of 2010 resulted in transformative reforms to school lunch and breakfast programs: more fruits, vegetables, and whole grains; less refined grains, sodium, calories, and saturated fats. Following the implementation of these stronger nutrition standards, studies consistently document significant improvements in the nutritional quality of school meals [6,7]. School lunches became 41% healthier and school breakfasts became 44% healthier, as measured by the Healthy Eating Index score [8].

### 1.3. Positive Effects of Participating in School Meals

Post-HHFKA research further highlights the benefits of healthier school meals. Nationally representative studies show that schools offering higher nutritional quality lunches are associated with higher rates of participation in school lunch programs—61% on average compared to 50% for schools with the least healthy meals [3]. In addition, students in food-insecure and marginally secure households were more likely to participate in school meal programs and receive more of their daily energy needs from school meals compared to students in food-secure households [9]. The nutritional quality of school lunches is now consistent across all school poverty levels and racial/ethnic composition, thus reducing disparities across socio-economic status, race, and ethnicity [10]. This reveals the positive impact a single federal policy can have on children’s diets and overall health [11].

### 1.4. Remaining Challenges and Recent Policy Concerns

Despite this considerable progress, there is still room for further improvements in the nutritional quality of and access to school meals. For example, historically, there has not been an added sugar standard for reimbursable school meals. During the 2014–2015 school year, nearly all school breakfasts (92%) had excessive amounts of sugar, while almost three-quarters of lunches (69%) exceeded the Dietary Guidelines for Americans (DGA) recommendations [12]. Flavored milk was the leading source of added sugars in school meals. In addition, given that ‘hunger’ is the most commonly reported reason for participation in school meal programs, federal, state, and local policies must prioritize expanding access to school meals [3].

Unfortunately, recent U.S. policy actions have limited access to necessary safety net programs, which impacts eligibility for nutrition programs such as school meals. This commentary discusses the importance of federal school meal programs on children’s nutrition and health, and highlights the implications of recent U.S. federal policy changes on school meals.

## 2. Recent U.S. Policy Actions

Numerous U.S. policy actions introduced or enacted in 2025 will impact the foods served in schools and access to school meal programs during the upcoming school year. These include cuts to local food programs, efforts to reduce ultra-processed foods in schools, new added sugar standards to be implemented in the 2025–2026 school year, and new eligibility restrictions for other federal programs, which will impact students’ and schools’ participation in universal free school meal policy options.

### 2.1. Changes Impacting Foods Served in Schools

#### 2.1.1. Cuts to the Local Food Programs

About 75% of U.S. school food authorities (i.e., districts) participate in farm-to-school activities [13]. Participating in farm-to-school activities improves acceptance of and participation in school meals, increases consumption of fruits and vegetables at school, and supports local farmers [14]. Funding for farm-to-school programs has grown significantly since the early 2000s with many states investing funds to support local purchasing, and the establishment of a competitive grants program at the United States Department of Agriculture (USDA) as part of the 2010 Healthy, Hunger-Free Kids Act.

Additional funding for farm-to-school programs was provided to schools via a COVID-19 relief program, The Local Food For Schools Cooperative Agreement Program. Established in December 2021, this program supports regional food systems by providing funding to state agencies to purchase local, unprocessed, or minimally processed foods from farmers, ranchers, and small businesses to be served in the SBP and NSLP [15]. An additional $660 million was committed to the Local Food for Schools Cooperative Agreement program in October 2024 to help schools and childcare sites continue to purchase local foods over the next 3 years. Yet, in March 2025, despite already having contracts signed, USDA announced that they were terminating this new funding [16].

Local food programs such as these are influential in building the local food system by providing direct financial support to local farmers while feeding school-age children healthy, fresh food. Cuts to this program will impact schools’ ability to afford and provide locally grown food and are also likely to have negative effects on the local food system and rural economies.

#### 2.1.2. Ultra-Processed Foods in School Meals

While the nutritional quality of school meals improved following the 2010 HHFKA, concerns remain regarding the level of processing of the foods served and sold in schools. In fact, most schools across the U.S. regularly serve processed and ultra-processed convenience foods, including pre-portioned heat and serve items and/or quick preparation foods as part of breakfast and lunch [17]. A growing body of evidence linking consumption of ultra-processed foods (UPF), which often include toxic chemicals and additives, to adverse health effects, such as cardiometabolic, mental disorders, and mortality outcomes, has resulted in the topic gaining national attention in recent years [18]. Especially concerning for school-age children, nationally representative data show that the proportion of calories from UPF among children and adolescents has significantly increased and now comprises a majority of their total energy intake (57.9%) [19,20]. This has led many policymakers to focus on school meals as a mechanism for reducing consumption of UPF.

An evidence-based, federal definition of ultra-processed foods does not currently exist, complicating both research and U.S. policy regulation. In summer 2025, the U.S. Food and Drug Administration requested input on a definition for ultra-processed foods [21]. Otherwise, little action to date has occurred on the federal level. State action, however, has increased significantly in 2025. Numerous states have passed bans on dyes and additives in school, including Arizona, Arkansas, Utah, Louisiana, and West Virginia, with some bans going into effect as early as the 2026–2027 school year. California is the first state to define ultra-processed foods and move forward with banning them from the food supply, including in school meals. Bans on ultra-processed foods have the potential to improve the nutritional quality of school meals by increasing the availability of whole, healthy foods, such as fruits and vegetables, while eliminating (or reducing) frozen and pre-packaged meals like pizza, chips, and cereals.

#### 2.1.3. Added Sugar Standards

Beginning in the 2025–2026 school year, U.S. reimbursable school meals are required to meet added sugar standards for the first time using a phased approach [22]. Starting 1 July 2025, schools must implement product-based limits for breakfast cereals, yogurt, and flavored milk. These standards specify that breakfast cereals may have no more than 6 g of added sugar per dry ounce, yogurt may have no more than 12 g of added sugars per 6 ounces, and flavored milk may have no more than 10 g of added sugars per 8 fluid ounces. By school year 2027–2028, schools will be required to implement weekly dietary limits on added sugars to less than 10 percent of calories across the week in the school lunch and breakfast programs. These added sugar limits better align school meal nutrition standards with the 2020 Dietary Guidelines for Americans and will continue to improve the nutritional quality of foods and beverages served.

The 2025 Dietary Guidelines for Americans are expected to be released in fall 2025. It is unclear if the final guidelines will align with the Scientific Report of the 2025–2030 Dietary Guidelines Advisory Committee, which recommends that added sugars should be limited to less than 10 percent of calories per day starting at age 2 [23]. If they do not, there will be significant implications for schools as the nutrition standards for school meals are required by law to align with the U.S. Dietary Guidelines.

### 2.2. Recent Policy Actions Impacting Program Access

#### 2.2.1. The One Big Beautiful Bill Act

The “One Big Beautiful Bill Act” (OBB) is a U.S. federal reconciliation bill that was signed into law on 4 July 2025 [24]. Among other provisions, the bill makes significant cuts to funding for federal assistance programs and changes eligibility requirements to limit participation in the Supplemental Nutrition Assistance Program (SNAP), also known as food stamps, and Medicaid, which provides needs-based health coverage in the U.S. This stands to impact school meal program participation via a process known as school meals direct certification. Historically, students were automatically enrolled in free or reduced-price school meal programs if they also participated in SNAP, Medicaid, or TANF (Temporary Assistance for Needy Families). This direct certification process eliminates the need for families to complete separate applications for school meal programs, streamlining the process and ensuring more eligible children receive access to vital nutrition programs [25].

SNAP is administered by the United States Department of Agriculture (USDA) and provides financial benefits to households with low incomes to supplement their grocery budgets, allowing them to afford nutritious foods. Several SNAP eligibility requirements changed in the OBB. Most notable for school meals is the SNAP work requirement exemption for parents with children, which will now only apply to parents with children aged 14 and younger. Previously, this exemption was granted to parents with children ages 18 and younger. This means many high-school children are likely to lose access to SNAP benefits and, as a result, school meals. Individuals aged 18–54 who are able to work are also required to work 80 h per month. Work may include community service, an educational program, and/or a “work program.” In addition, the OBB eliminates work requirement exclusions for individuals who are homeless, veterans, or individuals in foster care. Many individuals classified as non-U.S. citizens under federal law are no longer eligible for SNAP. The OBB also increases the financial burden for states via increases in the required state-level contribution to SNAP administrative costs. It is unclear whether this increased state-level contribution, which will be significant for many states, will result in fewer states operating the SNAP program.

Provisions in the OBB also make Medicaid eligibility more difficult. First, the legislation imposes stricter work requirements. To remain eligible, individuals must either work, volunteer, or participate in a form of education for at least 80 h per month. Medicaid eligibility is now required to be redetermined every 6 months, as opposed to 12 months, with more frequent address and eligibility checks. Research has demonstrated that individuals relying on Medicaid are more likely to experience income fluctuations due to seasonal or hourly positions as well as other unpredictable life changes, such as moving, shifts in who is living in the household, and difficulty navigating the administrative process (i.e., notifications of renewals and deadlines). These commonly experienced fluctuations are not as great a concern when eligibility is determined based on a 12-month window, but narrowing the window to 6 months will make it more difficult for individuals to receive the coverage they need.

#### 2.2.2. Universal Free School Meals and Community Eligibility Provision

The momentum for universal free school meals began during the COVID-19 pandemic when waivers authorized by USDA allowed all schools in the United States to offer free school meals to every child, regardless of income. While this national policy was de-implemented during the 2022–2023 school year, nine states currently have permanent legislation to offer free school meals to all students regardless of household income, with many more states currently planning and negotiating free school meal bills. Universal free school meals increase school meal participation, improve diet quality and food security, and can even improve academic performance and attendance [25]. Offering universal free school meals at the federal or state level is the ideal policy option to have the greatest impact; there are other mechanisms for schools to offer free meals at the local level.

The Community Eligibility Provision (CEP) is a meal service option that allows schools in high-poverty areas to provide free meals to all students. Schools are eligible for CEP if 25% or more of their students are determined eligible for free meals through direct certification. CEP increases student participation and leads to improvements in health, attendance, and academic outcomes [26]. Additionally, emerging evidence suggests that participating in CEP has positive economic impacts on families by reducing household grocery spending [27]. The proportion of schools participating in CEP has significantly increased since its adoption in 2014, from 14,184 schools to 54,234 schools in 2024 [28]. The recent increase in CEP participation rates can largely be attributed to Medicaid Direct Certification, which started in school year 2023–2024. By the 2024–2025 school year, 44 states used Medicaid and household income to directly certify children for free school meals.

USDA currently reimburses schools participating in CEP using a formula calculation. The higher the percentage of students eligible through direct certification (also called Identified Student Percentage—ISP), the higher the reimbursement [29]. Therefore, the percentage of students eligible through direct certification affects the likelihood of a school participating in CEP, and it also affects the amount of money a school is reimbursed for the meals provided. If fewer students are directly certified due to stricter Medicaid and SNAP eligibility rules, it will be harder for schools to qualify for CEP, thereby limiting access to free and reduced-price meals to entire schools and student populations [29]. Additionally, if schools do not receive adequate reimbursement, they may be inclined not to participate in the school meal programs altogether. This would have devastating impacts on students’ nutrition, food security, and academic success nationwide.

## 3. Implications for School Meals

When a child is automatically enrolled in school meals via direct certification, families are not required to fill out multiple, separate applications, which is commonly cited as a barrier to participating in school meal programs. Some states utilize direct certification for additional programs, such as summer-EBT, which provides families with money for food throughout the summer months when school is not in session.

### 3.1. School Meal Participation

With restricted eligibility for SNAP, fewer students are expected to be directly certified for school meal programs. This may lead to fewer students participating in the program, and delays in submitting and approving applications for free- and reduced-price meals as school district and state agency staff will have a much larger volume of applications to review.

Participating in school meals improves diet quality, food security, and academic performance [25]. School meals currently reach the students who need them most. Children in food insecure and marginally secure households have higher participation rates in school meal programs compared to children in food secure households [9]. Moreover, students participating in school meals consume healthier lunches compared to students who bring food from home [6].

Any delay in enrolling students in free or reduced-price meals could prevent a child from receiving a meal. This will disproportionately impact specific student populations who are now ineligible for SNAP, like Cuban and Haitian children who are not citizens under federal law and children who entered the U.S. under asylum and refugee laws or based on urgent humanitarian reasons (e.g., survivors of domestic violence or human trafficking) [12].

### 3.2. Budgets and Operations

The increased administrative burden of these policy changes does not end with having to review and approve more school meal program applications. School meal participation is directly tied to school meal revenue, and with fewer participating students, schools will have fewer resources to support programs [29]. Even before recent policy changes, many schools across the U.S. reported challenges of insufficient funding, including for purchasing foods to serve in school meals, recruiting and retaining food service staff, and to support necessary kitchen equipment, kitchen facilities, and food storage [17]. This cost burden is only going to grow as participation shrinks and funding to support food purchases—like the local foods program—go into effect.

While more schools have been moving to scratch cooking and serving healthier foods as required by the 2010 HHFKA, these efforts to improve the healthfulness of school meals are also likely to be dampened by recent policy changes. With less incoming revenue, there will be no extra funds to purchase new kitchen equipment or train staff on scratch cooking. Even in the best of times, when school food service operations are getting fully reimbursed for every free meal served, this amounts to only $4.16 per meal during the current school year [30]. School food operations are already doing an incredible amount of work to meet nutrition guidelines without going into debt; recent cuts will only make their jobs more difficult.

## 4. Conclusions

Recent cuts to federal programs will have a trickle-down effect on children’s ability to receive nutritious school meals at low or no cost. As previously described, direct certification allows children to be automatically enrolled in free school meals. With fewer students now eligible for SNAP, there are fewer students receiving school meals through direct certification, now placing additional administrative burden on parents, caregivers, and ultimately on school staff. School nutrition staff will now be responsible for processing more applications and navigating increased challenges, especially with potential financial losses from key programs like CEP and the Local Food for Schools Cooperative Agreement.

School meal access for students across the country is being threatened by budget cuts. Advocacy efforts are needed to preserve and strengthen USDA school meals and continue to reduce food insecurity. Research is also needed to assess the impacts of these cuts on participation rates, CEP participation rates, school funding, and administrative burden. Large-scale, national evaluation efforts like the School Nutrition and Meal Cost Study offer invaluable insights into the successes and challenges of school meal programs. These much-needed resources have been significantly compromised by recent staffing cuts to USDA and substantial funding cuts to research across all federal agencies, including discontinued research efforts by NIH and national data surveillance efforts. These recent policy actions have the potential to significantly impact child hunger and nutrition by limiting student participation, program eligibility, and increasing administrative burden in the nation’s second-largest nutrition program.

## Data Availability

No new data were created or analyzed in this study. Data sharing is not applicable to this article.

## Abbreviations

SBP	School Breakfast Program
NSLP	National School Lunch Program
HHFKA	Healthy, Hunger-Free Kids Act
DGA	Dietary Guidelines for Americans
USDA	United States Department of Agriculture
UPF	Ultra-processed Foods
OBB	One Big Beautiful Bill Act
SNAP	Supplemental Nutrition Assistance Program
TANF	Temporary Assistance for Needy Families
CEP	Community Eligibility Provision
ISP	Identified Student Percentage
